# A Macro-Pulse Photon Counting Lidar for Long-Range High-Speed Moving Target Detection

**DOI:** 10.3390/s20082204

**Published:** 2020-04-13

**Authors:** Yang Yu, Bo Liu, Zhen Chen, ZhiKang Li

**Affiliations:** 1 Institute of Optics and Electronics, Chinese Academy of Sciences, Chengdu 610209, China; yuyang215@mails.ucas.edu.cn (Y.Y.);; 2 University of Chinese Academy of Sciences, Beijing 100049, China; 3 Key laboratory of space optoelectronic precision measurement technology, CAS, Chengdu 610209, China

**Keywords:** Lidar, photon counting, macro pulse, pulse accumulation

## Abstract

A macro-pulse photon counting Lidar is described in this paper, which was designed to implement long-range and high-speed moving target detection. The ToF extraction method for the macro-pulse photon counting Lidar system is proposed. The performance of the macro pulse method and the traditional pulse accumulation method were compared in theory and simulation experiments. The results showed that the performance of the macro-pulse method was obviously better than that of the pulse accumulation method. At the same time, a laboratory verification platform for long range and high-speed moving targets was built. The experimental results were highly consistent with the theoretical and simulation results. This proved that the macro pulse photon counting Lidar is an effective method to measure long range high-speed moving targets.

## 1. Introduction

The photon counting Lidar (light detection and ranging) system is considered to be a promising sensor for ranging or imaging because of its high sensitivity, which can respond to a single photon, and provide very precise photon arrival time [1,2,3,4]. More and more researchers are interested in it, and it is widely used in the field of space exploration, especially in space debris identification and removal, space-borne topographic mapping, autonomous rendezvous and docking, and so on [5,6,7]. In these applications, there is a common phenomenon that there is relative motion between the Lidar platform and targets. This relative motion can seriously affect the detection performance of the Lidar system, which requires the Lidar system to have rapid detection capabilities.

Compared with linear mode Lidar, photon counting Lidar needs multi-period statistics to extract the target position. It is a statistical sampling technique and needs to accumulate enough photon events to establish the statistical histogram. This is also its main disadvantage, especially when there is relative motion between the Lidar system and the target. High-speed moving targets or Lidar platforms may cause it to be impossible to extract the range from the statistical histogram at all, since they are not clustered enough in the time domain. Therefore, how to effectively reduce the measurement time of photon counting Lidar is an urgent problem that needs to be solved.

Research groups have proposed the use of a high repetition rate laser source (tens of MHz) to reduce the data acquisition time [8]. However, it would greatly reduce the maximal unambiguous distance. Therefore, there is a contradiction between the long unambiguous distance and the short acquisition time of the traditional pulse accumulation method. In other words, it is difficult to employ for long-range and high-speed moving target detection. Du [9] and Liang [10] demonstrated a high-speed photon-counting laser ranging system with laser pulses of multiple repetition rates to extend the unambiguous range. This is an effective method to realize long-range detection. Jonsson [11] realized the reconstruction of the trajectory of a moving cooperative target.

In order to reduce the measurement time, a pseudo-random coded photon counting ranging method has been proposed, which is a borrowed idea from spread spectrum communication [12,13]. In 1983, Takeuchi et al. took a random phase code modulate continuous-wave laser with an external optical modulator, as a transmitting laser source in a laser ranging system [14]. Hiskett [15], Krichel [16], and Ullrich [17] used a finite non-periodic pulse train or pulse-position modulation technique to resolve range ambiguity. Zhang realized the detection of non-cooperative in 1.2 km [18]. Yang realized high spatial resolution detection using a high speed pseudo-random modulation fiber laser ranging system [19]. However, in the pseudo-random coding method, the laser diode is usually used to generate the high frequency pulse sequence, which is difficult to obtain with the high peak power pulse laser. Therefore, it is not suitable for the field of space exploration of 100 km or even further.

In order to solve these problems, we proposed using macro-pulse photon counting Lidar for long-range and high-speed moving target detection. Compared to the pulse accumulation method, the unambiguous distance of the macro-pulse method is determined by the macro pulse period, and the measurement time is limited by the total interval of the sub-pulses. Therefore, it can achieve fast moving target detection. Unlike the pseudo-random coded method, the macro-pulse method can emit a macro-pulse periodically. Therefore, a solid-state laser can be used to obtain higher laser power to realize long-range detection. Therefore, the macro-pulse photon counting Lidar is an effective method to resolve the problem of long-range and high-speed moving target detection.

## 2. System Description

### 2.1. System Structure of the Macro-Pulse Photon Counting Lidar

**Figure 1:**
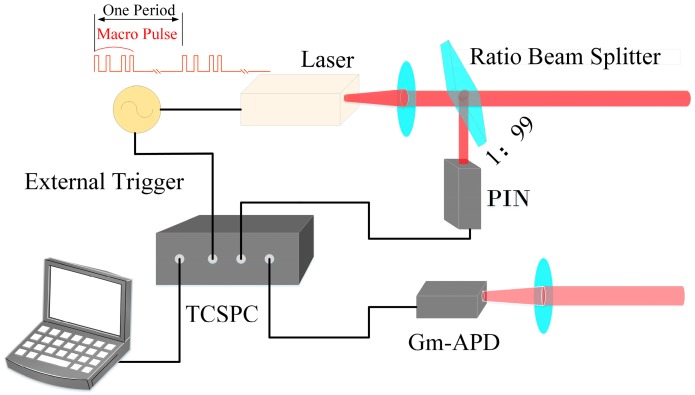
Schematic of the macro-pulse photon counting Lidar system, TCSPC (time-correlated single-photon counting), Gm-APD (Geiger-mode avalanche photodiode).

As shown in Figure 1, the Lidar emits a periodic pulse train, which is called a macro-pulse. The system is synchronized by an external clock. The output macro-pulse is split into two branches by a ratio beam splitter: one small part of the energy is detected by a PIN (p-type, intrinsic, n-type diode) detector, which is used as the transmitted reference signal, and most of the energy transmits to the target. The signal scattered by the target is received by the optical system, detected by the Gm-APD (Geiger-mode avalanche photodiode), and used as the received echo signal. In this macro-pulse Lidar system, any two sub-pulses have unequal pulse intervals, which can effectively avoid the range ambiguity.

### 2.2. The Ranging Principle of the Macro-Pulse Photon Counting Lidar System

**Figure 2:**
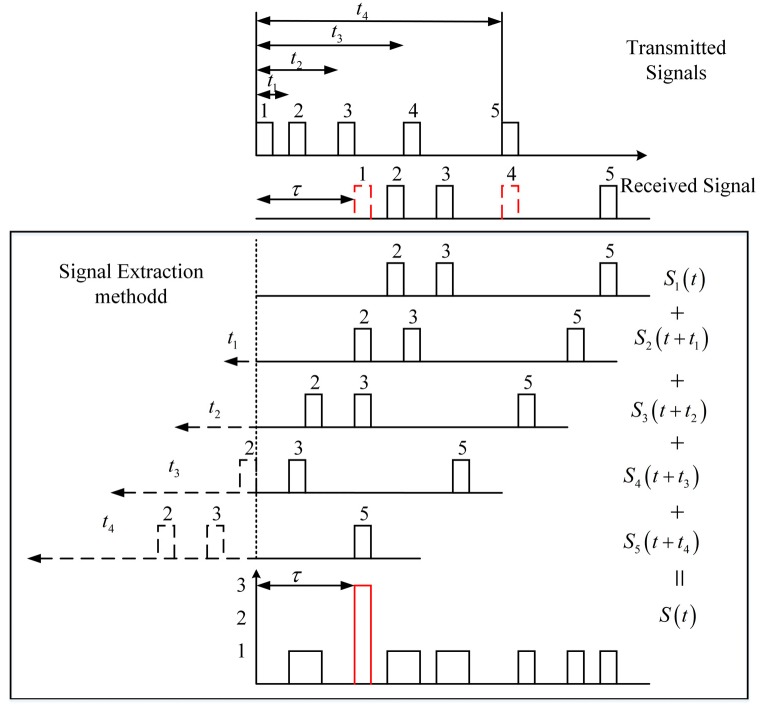
Shift pulse accumulation method.

The ToF (time of flight) extracted by the shift pulse accumulation method has no requirement for received signal, but it is necessary to know the sub-pulse intervals of the transmitted signal. In order to meet this requirement, we used the PIN detector to detect the emission sequence. The time intervals of the sub-pulse are measured and maintained as a known parameter. In one period, any two sub-pulses of the macro-pulse have unequal pulse intervals. As shown in Figure 2, in one period, the echo signal detected by the Gm-APD sequentially shifted the pulse intervals of the transmitted sub-pulses. The shifted echo signals are stored and accumulated. The red dotted line indicates that the transmitted sub-pulse has not been detected. The black dotted line represents invalid data in the shift pulse accumulation method due to compensation for time delay. The peak position (red solid line) corresponds to the ToF (τ) of the macro-pulse. In other words, because the delay time of each sub-pulse compared to the first sub-pulse is known, the ToF of the macro-pulse is equal to the ToF of the sub-pulses after their delay time is compensated. By compensating the delay of the sub-pulses and accumulating the sub-pulses, the SNR (signal-to-noise ratio) can be effectively improved, and the ToF of the macro-pulse can be highlighted.

After shifting the sub-pulses, the echo position of the target can be obtained by accumulating them. The pulse accumulating process after the sub-pulse shift is consistent with that of the pulse accumulation method. The purpose of sub-pulse accumulation is also to improve the SNR of the system.

**Figure 3:**
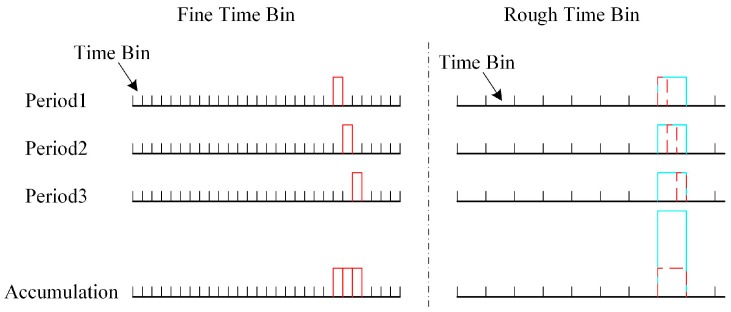
The effect of target motion on pulse accumulation in the case of fine time bin and rough time bin.

However, when the target moves, multiple pulses actually detect the target at different positions, so the ToF of each pulse is different. As shown in Figure 3, in the case of the fine time bin, due to the motion of the target, the echo signals of three periods are located at different time bins. At this time, although three periods accumulated, it was difficult to highlight the echo position of the target. To solve this problem, we needed to decrease the time resolution of the system and make the target nearly stationary at a low timing resolution. Then, the echo position of the target can be highlighted by accumulating multiple pulses. As shown in Figure 3, in the case of a rough time bin, even though the ToF of the target is different, the motion of the target is neglected because of the low timing resolution. At this time, the position of the target can be highlighted by accumulating multiple periodic pulses.

For the macro-pulse method, the setting of the rough time bin is related to the total sub-pulse interval, pulse width, and the velocity of the target. For the pulse accumulation method, it is related to the PRF (pulse repetition frequency), accumulated pulse number, pulse width, and the velocity of the target.

Assuming that the target relative to the Lidar system has a radial velocity $v_{t}$, the total interval of *N* sub-pulses is $t_{M}$ and the pulse width is $t_{fwhm}$, the rough time bin of macro-pulse method should not be less than $t_{bin\_M}$

(1) $$t_{bin\_M}=\frac{2t_{M}v_{t}}{c}+t_{fwhm}$$

where *c* is the speed of light.

The rough time bin of the pulse accumulation method should not be less than $t_{bin\_A}$

(2) $$t_{bin\_A}=\frac{2\frac{N}{PRF}v_{t}}{c}+t_{fwhm}$$

where *N* is the number of accumulation pulses and *PRF* is the pulse repetition frequency.

The total interval of *N* sub-pulses in the macro-pulse method is much smaller than that of the *N* pulses in the pulse accumulation method. When measuring a moving target, the macro-pulse method can choose a much smaller time bin than the pulse accumulation method.

It should be pointed out that in order to reduce the impact of range walk error [20], the setting of time bin includes the pulse width.

### 2.3. False Alarm Probability and Detection Probability

The excitation of Gm-APD depends on two consecutive random processes: photons entering the Gm-APD are converted to primary electrons, then primary electrons generate an avalanche up to a large current surge. For Gm-APD, noise primary electrons are mainly produced by two parts: (1) background photons, and (2) dark current generated by internal thermal noise. The avalanche is initiated by one or more primary electrons and results in a random number of charge carriers. When the number of primary electrons is few, the detection model of the Gm-APD approximately follows Poisson statistics [21]. For the Poisson process, the noise probability density function (PDF) describes the probability that *k* noise events occur. This probability of *k* noise avalanche events occurring in a time bin is given by

(3) $$p\left(k\right)=\frac{1}{k!}m_{n}{}^{k}\exp\left(-m_{n}\right)$$

where $m_{n}\left(t,t+t_{bin}\right)=\int_{t}^{t+t_{bin}}\psi_{n}\left(t\right)dt$ is noise primary electrons number in a time bin; $\psi_{n}$ is the noise primary electrons flux; and *k* is the number of generating avalanche. As shown in Equation (4), the probability that zero avalanche events occurs is $p\left(k=0\right)=\exp\left(-m_{n}\right)$. The Gm-APD is triggered when at least one avalanche event occurs. Thus, the noise trigger probability of a time bin is

(4) $$P_{n}\left(k>0\right)=1-\exp\left(-m_{n}\right)=1-\exp\left(-\int_{t}^{t+t_{bin}}\psi_{n}dt\right)$$

Assume that there are *N* sub-pulses in the macro-pulse, and *n* (1≤n≤N) is the threshold of the recognition target. When false alarms occur under the detection mode of the macro-pulse, it is indicated that at least *n* noise counts are generated with the same pulse intervals of the sub-pulses. Since the background light noise and dark count noise are obedient to uniform distribution, the false alarm probability of the macro-pulse method ($P_{FM}$) in a time bin can be written as

(5) $$P_{FM}\left(n\leq N\right)=\sum_{i=n}^{N}C_{N}^{i}P_{NM}^{i}{\left(1-P_{NM}\right)}^{N-i},C_{N}^{i}=\frac{N!}{i!\left(N-i\right)!}$$

(6) $$P_{NM}=\exp\left(-\int_{t-t_{d}}^{t}\psi_{n}\left(t\right)dt\right)\cdot \left(1-\exp\left(-\int_{t}^{t+t_{bin\_M}}\psi_{n}dt\right)\right)$$

where $P_{NM}$ is the noise detection probability of a time bin considering the detector’s dead time in the macro-pulse method. $t_{d}$ is the dead time of the Gm-APD, and *n* is the threshold of detection.

For the pulse accumulation method, the occurrence of false alarm events indicates that at least *n* noise counts appear at the same time bin of multiple periods. The false alarm probability of the pulse accumulation method ($P_{FA}$) can be written as

(7) $$P_{FA}\left(n\leq N\right)=\sum_{i=n}^{N}C_{N}^{i}P_{NA}^{i}{\left(1-P_{NA}\right)}^{N-i}$$

(8) $$P_{NA}=\exp\left(-\int_{t-t_{d}}^{t}\psi_{n}\left(t\right)dt\right)\cdot \left(1-\exp\left(-\int_{t}^{t+t_{bin\_A}}\psi_{n}dt\right)\right)$$

where $P_{NA}$ is the noise detection probability of a time bin considering the detector’s dead time in the pulse accumulation method.

**Figure 4:**
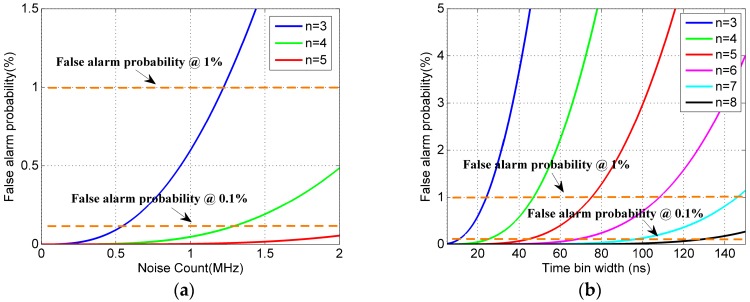
False alarm probability. (**a**) Influence of noise count on false alarm probability (*N* = 20, *t_d_* = 50 ns, *t_bin_* = 20 ns). (**b**) Influence of time bin width on false alarm probability (*N* = 20, *t_d_* = 50 ns, $\psi_{n}=1\;Mcps$).

Noise is one of the main factors affecting false alarm probability. It can be found from Figure 4a that when the time bin is determined, the false alarm probability increases exponentially with the increase in the noise count. In order to meet the requirement of not exceeding the maximum false alarm probability, the higher the noise count, the higher the threshold (*n*). Both the macro-pulse method and the pulse accumulation method follow this rule.

Comparing Equations (5)–(8), we can find that the main difference of false alarm probability between the two methods is the width of the rough time bin. As the noise is obedient to uniform distribution, the noise primary electrons in the rough time bin is positively correlated with the width of the rough time bin. As shown in Figure 4b, the false alarm probability increases exponentially with the increase of the time bin width. Under different time bin widths, in order to ensure that the maximum false alarm probability is not exceeded, the wider the time bin, the higher the signal recognition threshold (*n*). With the system parameters shown in Figure 4b, (*N* = 20, $t_{d}$ = 50 ns, $\psi_{n}=1\;Mcps$ (million count per second)), in order to ensure that the false alarm probability is less than 1%, when the time bin width is 40 ns, the threshold can be set to 4, while when the time bin width is 100 ns, the threshold should be set to 6.

The macro-pulse method can choose a narrower time bin than the pulse accumulation method. Thus, under the condition of the same noise count level, the macro-pulse method can select a smaller threshold. $n_{M}$ and $n_{A}$ represent the thresholds of the macro-pulse method and the pulse accumulation method, respectively. The detection probability ($P_{DM}$) of the macro-pulse method can be written as

(9) $$P_{DM}=\left(N\geq n_{M}\right)=\sum_{i=n_{M}}^{N}C_{N}^{i}P_{M}^{i}{\left(1-P_{M}\right)}^{N-i}$$

(10) $$P_{M}=\exp\left(-\int_{t-t_{d}}^{t}\psi_{n}\left(t\right)dt\right)\cdot \left(1-\exp\left(-m_{s}-m_{nM}\right)\right)$$

where $P_{M}$ is the detection probability of each sub-pulse in the macro-pulse method. $m_{nM}=\int_{t_{bin\_M}}\psi_{n}dt$ is the average noise primary electrons number per rough time bin in the macro-pulse method and the *m_s_* is the average signal primary electron number.

While the detection probability ($P_{DA}$) of the pulse accumulation method can be written as

(11) $$P_{DA}=\left(N\geq n_{A}\right)=\sum_{i=n_{A}}^{N}C_{N}^{i}P_{A}^{i}{\left(1-P_{A}\right)}^{N-i}$$

(12) $$P_{A}=\exp\left(-\int_{t-t_{d}}^{t}\psi_{n}\left(t\right)dt\right)\cdot \left(1-\exp\left(-m_{s}-m_{nA}\right)\right)$$

where $P_{A}$ is the detection probability of each pulse in the pulse accumulation method, $m_{nA}=\int_{t_{bin\_A}}\psi_{n}dt$ is the average noise primary electron number per rough time bin.

**Figure 5:**
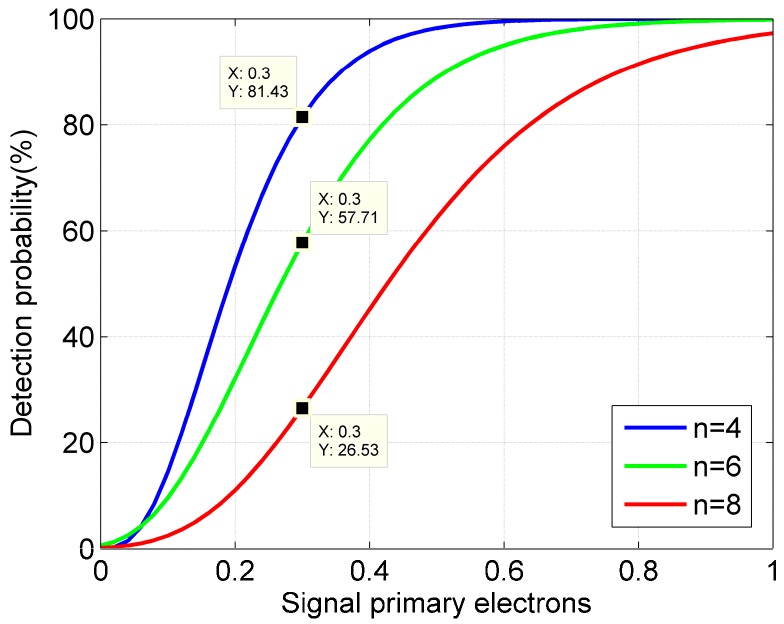
Influence of threshold on detection probability (*N* = 20, *t_d_* = 50 ns, $\psi_{n}=1\;Mcps$).

From Figure 5, it can be found that with the increase of the signal primary electrons, the detection probability increases gradually and tends to be saturated. At the same time, when the detection probability is unsaturated, the smaller the threshold, the higher the detection probability.

In general, according to Figure 4 and Figure 5, it can be seen that under the condition of the same maximum false alarm probability, the detection probability of the two methods gradually decreases as the width of the time bin increases. As the rough time bin of the macro-pulse method is smaller than that of the pulse accumulation method, the detection probability of the macro-pulse method is higher than that of the pulse accumulation method.

## 3. Simulation and Experiment

### 3.1. Simulation

In order to compare the performance of the macro-pulse method and pulse accumulation method for moving target detection, Monte Carlo simulation experiments were completed. The main simulation parameters are shown in Table 1. The simulation process is shown in Table 2.

In the simulation, the target was assumed to be 100 km away from the Lidar system and moved to the Lidar system with a velocity of 1500 m/s. The PRF of the pulse accumulation method was 1 kHz. When 20 pulses were accumulated, the target moved 30 m. According to Equation (1), the rough time bin width of the pulse accumulation method was set as 205 ns. However, in the macro-pulse method, 20 sub-pulses with unequal intervals were distributed within 1 ms, and the target moved 1.5 m. The target was nearly stationary. According to Equation (2), the rough time bin width of the macro-pulse method was set to 15 ns.

**Figure 6:**
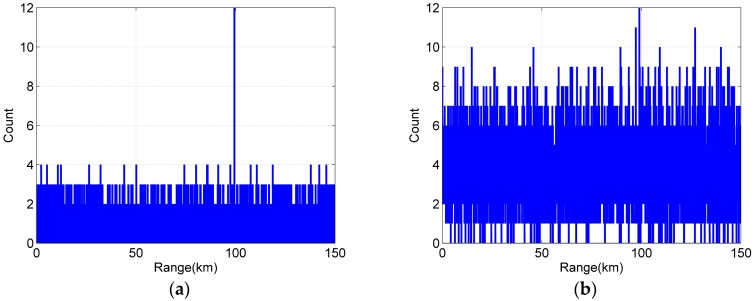
Histogram. (**a**) Macro pulse method (rough time bin 8 ns). (**b**) Pulse accumulation method (rough time bin 140 ns).

The pulse accumulation method used the 205 ns time bin to directly accumulate 20 periods to obtain the accumulation histogram. However, the macro-pulse method used the 15 ns time bin to obtain the accumulation histogram by the shift pulse accumulation method. The maximum count position of the histogram was taken as the target position for both methods. The histograms of both method are shown in Figure 6. It can be found that the noise count of the pulse accumulation method histogram was significantly higher than that of the macro-pulse method. This is because the false alarm probability increases with the increase in the time bin width. The rough time bin width of the pulse accumulation method is wider than that of the macro pulse method. Therefore, after multi-period accumulation, the pulse accumulation method will accumulate more noise counts than the macro-pulse method. As shown in Figure 6, the threshold value of the macro pulse method can be set to 5, while that of the pulse accumulation method needs to be set to 12.

**Figure 7:**
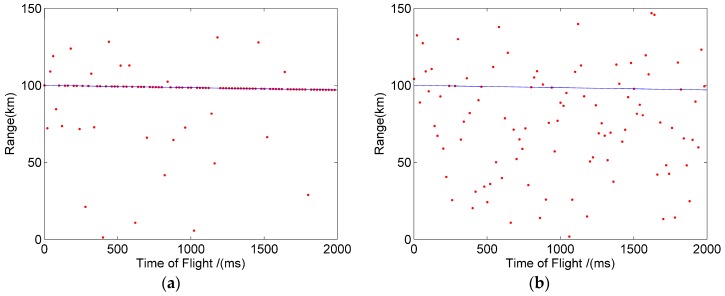
Target trajectory of the simulation. (**a**) Macro pulse method. (**b**) Pulse accumulation method.

In order to verify the feasibility of the method for a long-range high-speed moving target, we assumed that the target flies from 100 km to the Lidar system with a uniform speed (1500 m/s) and continued to detect the target for two seconds. In two seconds, the two methods can give 100 distance values of the target. Figure 7a,b show the moving trajectory of the target detected in one simulation, when the mean number of primary electrons is about 0.35. In Figure 7, the blue line represents the actual trajectory of the target, and the red point represents the position of the target detected by the macro-pulse method and the pulse accumulation method. The red dots on the blue line indicate that the target is correctly detected, while other red dots represent the target that is incorrectly identified. Comparing Figure 7a and b, it can be found that the detection probability of the macro-pulse method was significantly higher than that of the pulse accumulation method.

In order to compare the detection probability of the macro pulse method and the pulse accumulation method quantitatively, the above simulation process was repeated 50 times respectively under the three echo intensities, and the statistical results of the detection probability of the two methods are shown in Table 3. From Table 3, it can be found that the detection probability of the macro-pulse method is always much higher than that of the pulse accumulation method under the three echo signal intensities.

### 3.2. Experiment

**Figure 8:**
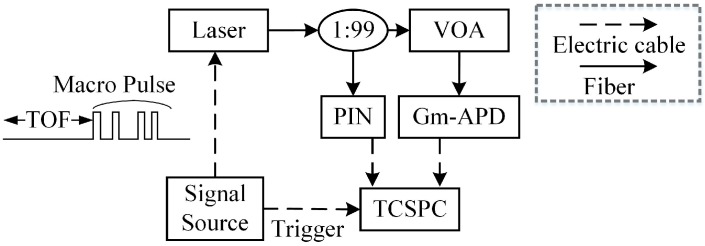
Experimental schematic diagram of high speed moving target detection.

In order to prove that the macro-pulse method is more suitable to detect moving targets than the pulse accumulation method, we built an equivalent moving target measurement platform. The experimental schematic is shown in Figure 8. The manufacturer, model, and main specifications of the equipment are shown in Table 4. The echo signal of a long-distance and high-speed moving target was generated by a 5G high bandwidth signal generator (AWG5200) and an externally modulated laser source. The core idea was to use the signal generator to construct the modulation signal including the ToF, then use the modulation signal to trigger the laser to generate the echo signal. In this way, the requirement of long distance is realized. In addition, the attenuation of distance to laser pulse energy should also be considered. Therefore, a VOA (adjustable optical attenuator) was added before the Gm-APD detector to simulate the echo signals of different intensities by adjusting the attenuation ratio of VOA. Thus, an equivalent detection system of a long-distance high-speed moving target was constructed. In practical application, the 1064 nm laser was selected from the point of view of long-distance detection requiring high-power laser. Therefore, we chose a near-infrared enhanced silicon-based Gm-APD as the detector, which is the most commonly used type of detector in the 1064 nm band.

**Figure 9:**
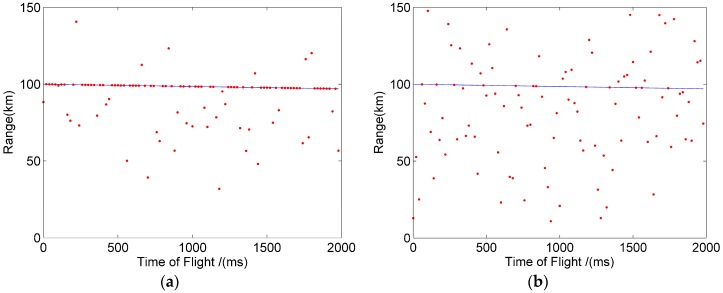
Target trajectory of experiment. (**a**) Macro pulse method. (**b**) Pulse accumulation method.

Just like the simulation experiment, it was assumed that the target flies from 100 km to the Lidar system at a speed of 1500 m/s. Figure 9a,b show the target detection trajectory of the macro pulse method and the pulse accumulation method when the mean signal primary electrons was 0.35, respectively. It can be seen that the detection probability of the macro pulse method was significantly higher than that of the pulse accumulation method, and the results were consistent with the simulation results.

By adjusting the attenuation ratio of the VOA, the detection performance of the two methods could be compared under different echo intensity (mean signal primary electron numbers were 0.35, 0.5, 0.75). Under the three echo intensities, the theoretical results, simulation results, and experimental results of the detection probability of the macro pulse method and the pulse accumulation method were comprehensively counted, as shown in Table 5. The signal recognition threshold value of the theoretical result, as shown in Figure 6, was set as 5 and 12 for the macro-pulse method and the pulse accumulation method, respectively. According to the statistical data in Table 5, it can be found that the theoretical results, simulation results, and experimental results were highly consistent; that is to say, the three different methods proved that the macro-pulse method has obvious advantages over the traditional pulse accumulation method in measuring long-distance high-speed moving targets.

## 4. Conclusions

A macro-pulse photon counting Lidar system for long-range high-speed target detection was proposed in this paper. The shift pulse accumulation method was proposed to extract the ToF of the macro pulse. In this Lidar system, unequal interval pulse trains are transmitted. The length of a pulse train is typically hundreds of microseconds or even hundreds of nanoseconds. During such short time intervals, the movement of the target can be ignored. Therefore, compared with the pulse accumulation method, the macro pulse method can choose a smaller rough time bin. The narrower the rough time bin, the lower the false alarm probability, and, the lower the false alarm probability of the single pulse, the smaller the noise count of the statistical histogram. Thus, the macro pulse method can select a smaller signal extraction threshold (*n*), improving the detection probability of the target. In conclusion, macro pulse photon counting Lidar is more suitable for high-speed moving target detection than pulse accumulation photon counting Lidar as the pulse intervals of sub-pulses are set to be not equal to avoid range ambiguity.

The performance of the macro pulse method and the pulse accumulation method were compared for long-range high-speed moving target detection. The simulation and experiment results were consistent with the theoretical analysis and showed that the macro-pulse method had a higher detection probability than the pulse accumulation method. A long-range high-speed moving target detection Lidar system was established with a high bandwidth signal generator and an externally modulated laser. Based on this experimental platform, we completed the principle verification. In general, we have proved that the macro pulse method has obvious advantages over the pulse accumulation method when detecting high-speed moving targets through theory, simulations, and experiments.

## Figures and Tables

**Table 1:** Main parameters of the Lidar system.

Parameter	Value
Velocity	1500 m/s
Pulse Width	4 ns
Mean signal primary electrons	0.3
Mean noise count	1 Mcps
Sub-pulse number (*N*)	20
Dead time	50 ns
Fine time bin	100 ps

**Table 2:** The simulation process.

1. Set high-speed moving target trajectory.
2. Generate noise and signal at fine time bin.
3. Determine the rough time bin and process the data in Step 2 with rough time bin.
4. Based on the Step 3, the pulse accumulation method directly accumulates *N* period, and the macro-pulse method accumulates *N* sub-pulses with the shift pulse accumulation method.
5. Determine the thresholds (*n*) of the two methods and extract the echo position.

**Table 3:** Detection probability statistics.

Mean Echo Primary Electron Number	Detection Probability (%)	
	Macro Pulse	Pulse Accumulation
0.35	67.6	13.4
0.5	94.0	29.4
0.75	99.4	59.4

**Table 4:** Main specifications of the equipment.

Equipment	Manufacturer	Model	Specification	
Laser	Connet Fiber Optics	VLSS-1064-M-PL	Wavelength	1064 nm
			Pulse width	4 ns
			Peak power	1 kW
Gm-APD	Excelitas Technologies	SPCM-NIR-10-FC	Dead time	35 ns
			Photon detection efficiency	2%
PIN	Thorlabs	APD310	Bandwidth	1 GHz
TCSPC	Siminics	FT1040	Time resolution	64 ps

**Table 5:** Detection probability of the macro pulse method and pulse accumulation method.

Mean Echo Primary Electron Number	Method	Detection Probability (%)		
		Theory	Simulation	Experiment
0.35	Macro Pulse	76.2	67.6	68.0
	Pulse Accumulation	7.4	13.4	9.5
0.5	Macro Pulse	94.4	94.0	90.2
	Pulse Accumulation	23.3	29.4	28.8
0.75	Macro Pulse	99.7	99.4	98.4
	Pulse Accumulation	60.0	59.4	57.4
